# Research on a Self-Powered Vibration Sensor for Coal Mine In Situ Stress Fracturing Drilling

**DOI:** 10.3390/mi17010131

**Published:** 2026-01-20

**Authors:** Jiangbin Liu, Mingzhong Li, Chuan Wu, Xianhong Shen, Yanjun Feng

**Affiliations:** 1 Coal Mining Branch, China Coal Research Institute, Beijing 100013, China; liujiangbin_1210@163.com; 2 Shaanxi Shaanxi Coal Caojiatan Mining Co., Ltd., Yulin 719100, China; 3 Beijing Tianma Intelligent Control Technology Co., Ltd., Beijing 100399, China; limingzhong_1210@163.com; 4 Faculty of Mechanical and Electronic Information, China University of Geosciences (Wuhan), Wuhan 430074, China; 15271506839@163.com; 5 China Coal Technology & Engineering Group Coal Mining Research Institute, Beijing 100013, China

**Keywords:** triboelectric nanogenerator, self-powered, vibration sensors, high reliability

## Abstract

In the process of in situ stress fracturing drilling in coal mines, obtaining downhole vibration data not only improves drilling efficiency but also plays a key role in ensuring operational safety. Nevertheless, the energy supply techniques used in current vibration detectors reduce operational performance and escalate excavation expenses. This research proposes a self-powered vibration sensor based on the triboelectric nanogenerator, designed for the operational environment of coal mine in situ stress fracturing drilling. It can simultaneously detect axial and lateral vibration frequencies, and the inclusion of redundant sensing units provides the sensor with high reliability. Experimental outcomes demonstrate that the device functions across a frequency span of 0 to 11 Hz, maintaining error margins for frequency and amplitude under 4%. Furthermore, it functions reliably in environments where temperatures are under 150 °C and humidity is under 90%, proving its strong resilience to environmental factors. In addition, the device possesses self-generating potential, achieving a maximum voltage of 68 V alongside an output current of 51 nA. When connected to a 6 × 10^7^ Ω load, the maximum output power can reach 3.8 × 10^−7^ W. Unlike traditional subsurface oscillation detectors, the proposed unit combines self-generation capabilities with highly reliable measurement characteristics, making it more suitable for practical drilling needs.

## 1. Introduction

During coal mining, high in situ stress concentrations in underground roadways can easily trigger disasters such as rock bursts and coal gas outbursts [1,2]. Therefore, proactively conducting directional drilling for hydraulic fracturing can accurately transfer and reduce the accumulated stress energy in critical zones, fundamentally enhancing safety [3]. During directional drilling in underground coal mines, interaction between the rock strata and the drill bit generates vibrations. Such vibration data contains rich information about lithological properties, drill equipment condition, and operational performance [4,5]. Hence, acquiring and interpreting subsurface oscillation data precisely not only represents a crucial step in achieving the dynamic optimisation of drilling parameters but also an important way to build intelligent drilling systems. However, achieving long-term and reliable monitoring of downhole vibrations is not easy due to energy supply challenges. Currently, the power required by downhole sensors is primarily provided by battery packs installed downhole or by cables extending from ground level [6,7]. The battery-based power supply is constrained due to its finite energy density and life decay under harsh downhole conditions. Once the power is depleted, time-consuming and costly tripping operations are required to replace the power supply unit. This not only constrains drilling timeliness but may also lead to the loss of critical geological information. While a wireline power supply is capable of delivering a steady stream of energy, the expense associated with wired drill pipes is remarkably high, and the cable connectors face a risk of connection failure in complex downhole conditions, making reliability difficult to guarantee. Therefore, if a sensor has the function of generating its own power by utilising downhole working conditions, these issues can be successfully addressed.

An effective way to mitigate the above-mentioned issues is to employ triboelectric nanogenerators (TENGs). By combining contact electrification with electrostatic induction, TENGs are capable of transforming ambient kinetic power available within the surroundings to generate useful electric power and simultaneously sense physical signals, thus showing great promise for applications in energy collection and signal detection [8,9]. Regarding electrical power generation, TENG devices have been successfully used for scavenging biomechanical motion [10], airflow [11], ocean waves [12,13], and sound waves [14,15,16]. Given that the electrical output of a TENG typically appears as a sequence of pulses whose frequency characteristics are governed by the excitation conditions, TENG has also been widely applied in various sensor fields, such as tactile monitoring [17,18], airflow velocity detection [19], pressure sensing [20,21,22], thermal tracking [23,24], moisture analysis [25,26], torque measurement [27], gas detection [28,29], healthcare applications [30], and fluid flow assessment [31,32]. In particular, TENG also has applications in vibration measurement, including vibration measurement of offshore platforms [33], pipelines [34], and bridges [35,36]. However, these sensors have low redundancy, and once damaged, it is difficult for them to continue operating normally.

Based on such advantages, the present work develops a redundant sensor for vibration monitoring that is self-powered and designed for geological drilling applications, based on the TENG technology. In this design, the vibration of the drill string drives its internal nanomaterials to generate power through friction, thereby realising the self-powering capability. Simultaneously, it utilises the direct correlation between the frequency of the generated TENG output and that of the oscillations produced by the drill string in order to achieve synchronous measurement of the oscillation rate. However, it is crucial to understand that the method proposed in this study is a new measurement technique that simultaneously measures vibration and converts downhole vibrational energy into electricity. The processing of the sensor’s output signal, however, still requires the development of subsequent processing circuits.

## 2. Structure and Working Principle

**Figure 1:**
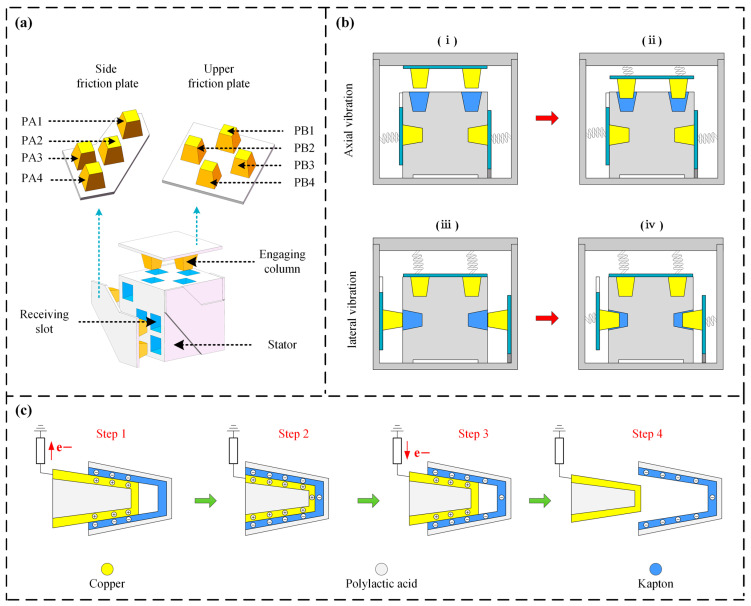
Illustration of the structural design, operational mechanism, and processing steps for the sensor. (**a**) Structural configuration of the sensor; (**b**) operational mechanism of the sensor; (**c**) illustration of the triboelectric signal generation mechanism.

Figure 1a displays the structural layout of the proposed redundant self-generating vibration sensor. This device is mainly composed of a fixed stator together with movable triboelectric units that are arranged to sense both the transverse vibrations of the drill string and axial vibrations. The frustum structure was chosen to take advantage of its self-guiding effect, which reduces the risk of mechanical jamming caused by misalignment during reciprocating motion. The lateral vibration module consists of four side friction plates, each carrying four meshing posts whose surfaces are covered with 30 mm × 30 mm × 0.05 mm layers of copper foil, acting as the friction layer. Wires are led out from each of these four meshing posts, serving as the four signal output channels for lateral vibration, named PA1, PA2, PA3, and PA4 (in order). Corresponding receiving slots are made on the sidewalls of the stator, and their inner walls are adhered with 30 mm × 30 mm × 0.05 mm Kapton as the friction layer. The axial vibration sensing unit has a similar structure to the lateral unit, consisting of an upper friction plate and four meshing posts below it. The receiving slots on top of the stator are also lined with Kapton, while the surfaces of the meshing posts are copper foil. Wires are led out from each meshing post, serving as signal outputs—named PB1, PB2, PB3, and PB4 (in order)—for vertical vibration monitoring. For the primary structural parts of the sensor, including the stator and friction plates, Polylactic Acid (PLA) is adopted and fabricated by means of 3D printing. During fabrication, the nozzle temperature is controlled at 210 °C, and the printing is performed with a layer height of 0.2 mm.

Figure 1b schematically depicts the operating mechanism of the sensor. This design can independently respond to and distinguish between axial and lateral external vibrations. As depicted in Figure 1(b(i),b(ii)), the operating mechanism under axial vibration is as follows: when the sensor is subjected to external axial vibration, the upper friction plate undergoes up-and-down reciprocating motion due to inertia, overcoming the preload force of the springs, while the lateral vibration module remains stationary. During this motion, the copper on the upper friction plate and the Kapton in the receiving slots on the stator top undergo periodic contact and separation, thereby generating a continuous triboelectric signal at the axial output terminals. The working process for lateral vibration is illustrated in Figure 1(b(ii),b(iv)). When the sensor is subjected to external vibration along a horizontal direction, the side friction plate in the corresponding direction undergoes horizontal reciprocating motion due to inertia, while the axial vibration module remains stationary. The side friction plate moves inward, and its surface copper electrode makes contact with the Kapton friction layer in the receiving slot of the stator sidewall, causing charge transfer. Afterwards, the spring drives it back to the original position, completing a contact–separation cycle. Therefore, by monitoring which specific side plate has a signal output, the direction of vibration can be determined, and by analyzing the signal frequency, measurement of axial and lateral vibrations can be achieved. The motion process is shown in Formula (1):

(1) $$m\frac{d^{2}x\left(t\right)}{{dt}^{2}}+c\frac{dx\left(t\right)}{dt}+kx\left(t\right)=F\left(t\right)$$

where m is effective mass (kg); c is the damping coefficient (N·s/m); k is the spring constant (N·m); $x\left(t\right)$ is displacement (m); and $F\left(t\right)$ is the external force (N).

Figure 1c presents a schematic illustration of how the triboelectric signal is generated. Since the fundamental mechanism for power generation in both axial and lateral vibration-sensing units is identical, the interaction of a single meshing post and a receiving slot is used as an exemplar for explanation. When vibration occurs, the process initially enters the phase shown in Figure 1c (Step 1). At this point, the copper foil on the meshing post surface starts to enter the receiving slot and makes contact with the Kapton friction layer. Due to contact electrification and the different abilities of the two materials to attract or release electrons, the Kapton gradually becomes negatively charged. To balance the potential, negative charges in the copper transfer outward, inducing an equivalent quantity of positive charge. Consequently, a transfer current flows through the loop, causing the voltage output to rise progressively. When the meshing post advances further to the position indicated in Figure 1c (Step 2), the charge transfer between the two surfaces is complete. The amount of charge on both friction layers reaches its maximum, and the output potential reaches its peak value. Afterwards, as the meshing post starts to separate, transitioning to the stage shown in Figure 1c (Step 3), the two friction layers gradually move apart. The electrostatic attraction of the accumulated negative charges upon the Kapton for the positive ones located on the copper foil diminishes, driving negative charges to move back to the conductive foil through the external loop. This produces a reverse flow of current within the loop, and the voltage signal drops accordingly. Ultimately, as illustrated in Figure 1c (Step 4), the meshing post and the receiving slot are completely separated, completing one power generation cycle. Each complete mechanical vibration cycle drives the triboelectric layer to complete one full contact–separation cycle, thereby generating a complete alternating current pulse. Therefore, the frequency of the output electrical signal can directly reflect the frequency of the source vibration. Thus, through employing a backend microcontroller to detect the frequency of such a TENG signal and by distinguishing which friction plate generated the signal, precise measurement of the vibration frequency and orientation can be achieved.

## 3. Sensor Performance Test

### 3.1. Experimental Design

**Figure 2:**
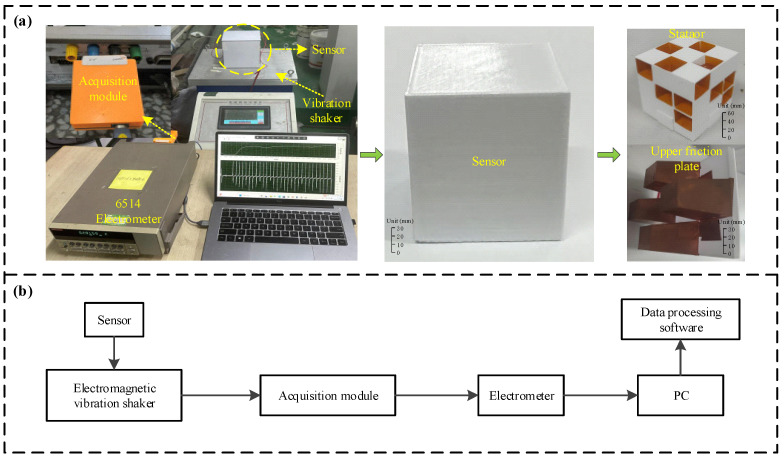
Illustration of the experimental arrangement. (**a**) Photo of the testing system; (**b**) schematic representation of the setup.

Experiments were conducted using the simulation setup shown in Figure 2a. The experimental apparatus comprised a vibration controller, an electromagnetic shaker, a data acquisition board, an electrometer, and a PC. The sensor was mounted onto the shaker, and the oscillation frequency and shaker amplitude were set by the vibration controller. As illustrated in Figure 2b, the raw outputs from the sensing unit were handled sequentially by the acquisition module and the electrometer before being sent to the PC. The host computer was installed with a LabVIEW-based program (13.0), which could display and store the sensor data in real time. The experiments were conducted to test the sensing performance of the device, as well as its redundancy, energy harvesting characteristics, and suitability to working conditions; further details will be described later in this paper.

### 3.2. Sensing Performance Test

**Figure 3:**
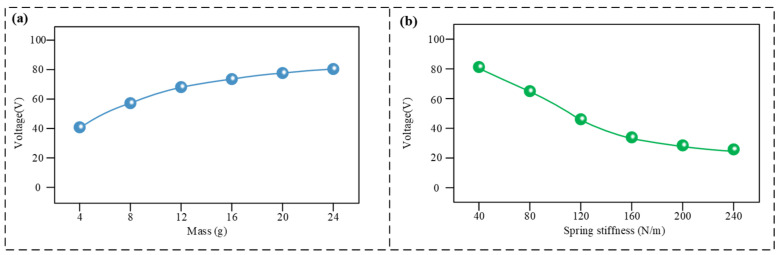
Sensor mechanical characteristic diagram. (**a**) Output voltage under different masses; (**b**) output voltage under different spring stiffnesses.

To further investigate the dynamic characteristics of the sensor, the influence of its mechanical parameters was analysed, with the results shown in Figure 3. As shown in Figure 3a, with the spring stiffness kept constant at 80 N/m, the output voltage exhibited a positive correlation with the moving mass. When the mass increased from 4 g to 24 g, the output voltage rose from approximately 40 V to about 80 V. This is because a larger mass generates greater inertial force, leading to more sufficient frictional contact. Figure 3b indicates that with the moving mass fixed at 12 g, when the spring stiffness increased from 40 N/m to 240 N/m, the output voltage decreased from approximately 82 V to about 25 V. This is because a stiffer spring restricts the amplitude of motion, leading to insufficient frictional contact and, consequently, reducing the output signal. Based on the actual downhole space and practical signal requirements, the final prototype was designed with a moving mass of 12 g and a spring stiffness of 80 N/m.

**Figure 4:**
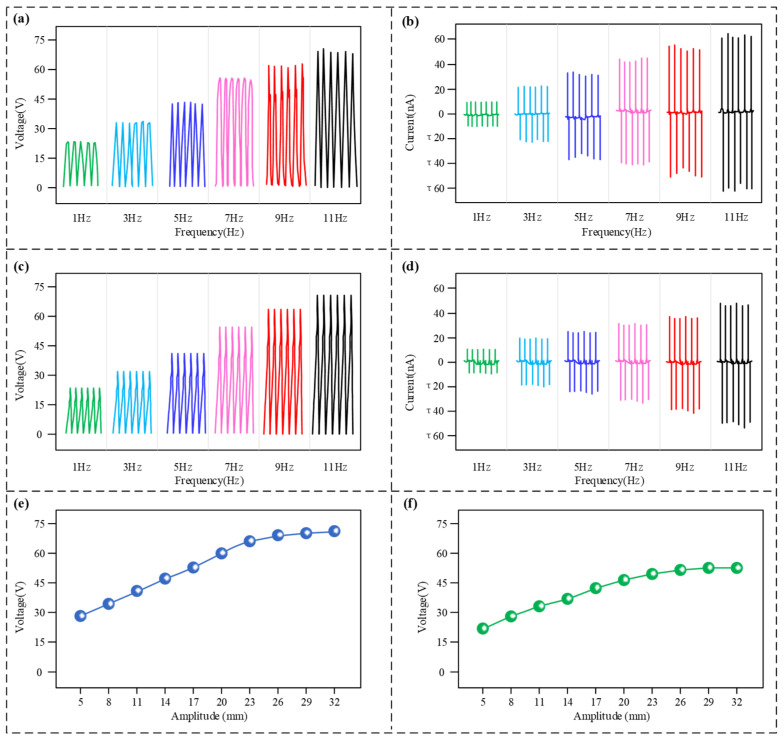
Sensing performance test results. (**a**) Voltage output signals from the lateral module under various frequencies; (**b**) generated current signals by the lateral module under various frequencies; (**c**) voltage output signals from the axial module at varying amplitudes; (**d**) current signals generated by the axial module at varying amplitudes; (**e**) lateral output voltage under different vibration amplitudes; (**f**) axial output voltage under different vibration amplitudes.

Further, the output performance exhibited by the sensor’s lateral and axial vibration modules was tested at different vibration frequencies, with the outcomes illustrated in Figure 4. As depicted in Figure 4a,b, the frequency response characteristics associated with the lateral vibration module were tested. Both the generated voltage and current signals manifest as AC pulse waveforms. As the vibration frequency increased within the range of 1 Hz to 11 Hz, the magnitudes of both the potential and current showed a distinct rising tendency. Upon the frequency attaining 11 Hz, the voltage generated by the detector peaked at 68 V, while the peak output current also reached 60 nA. Subsequently, the output characteristics of the axial vibration module were evaluated, with the results shown in Figure 4c,d. Its output behaviour demonstrated strong consistency with that of the lateral module, with the magnitude of both the generated electrical signals increasing steadily as the vibration frequency rose. With a frequency of vibration at 11 Hz, the peak voltage produced by the axial module could reach approximately 70 V, and the peak current was about 50 nA. Once the frequency of oscillation went above 11 Hz, the waveform generated by the device became disordered. This is mainly because, at higher frequencies, the restoring force of the spring is insufficient to push the moving unit back to the bottom of the slot before the start of the next cycle, thus preventing effective triboelectric contact. Therefore, the sensor’s effective frequency sensing range is defined as 0–11 Hz. This frequency range covers the main downhole vibration frequencies caused by drill string rotation and can be used, to some extent, to indicate the operational status of downhole drilling tools. The results above indicate that for both the lateral and axial modules, the signal output magnitude is positively correlated with the vibration frequency. This is because a higher vibration frequency implies a faster speed of contact and separation between the friction layers, which enhances the charge transfer efficiency during the triboelectrification process, resulting in an increased amplitude of the output electrical signal. Furthermore, considering the sensor’s high-voltage and low-current characteristics, the voltage signal will be used as the sensing signal in subsequent tests.

To investigate the sensor’s response characteristics to vibration intensity, we tested the variation in its output voltage with vibration amplitude at a fixed frequency of 10 Hz, as shown in Figure 4e,f. In the lateral vibration mode, as the vibration amplitude increased from 5 mm to 32 mm, the output voltage steadily increased from 28.1 V to 72.3 V. Similarly, in the axial vibration mode, the output voltage also increased from 21.5 V to 52.4 V as the amplitude increased from 5 mm to 32 mm. Both conditions demonstrate a clear positive correlation between the output voltage and the vibration amplitude.

### 3.3. Rotational Angle Measurement Experiments

**Figure 5:**
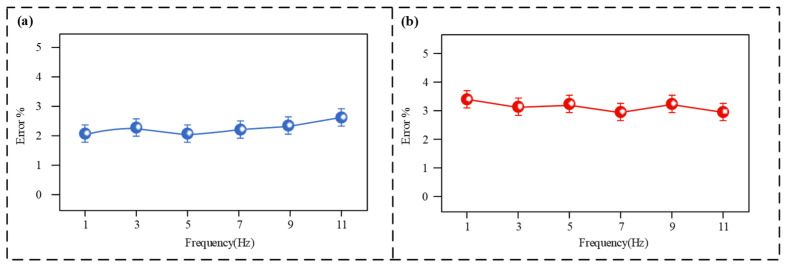
Measurement error results. (**a**) Measurement error of the lateral module under various frequency conditions; (**b**) error analysis regarding the axial direction module under various frequency conditions.

To evaluate the sensor’s accuracy, reliability, and other characteristics, we conducted 10,000 measurement tests for each type. Each friction plate of the sensor is equipped with four identical measurement units. Each unit provides redundancy for both lateral and axial vibration frequency measurements, ensuring the high reliability of the sensor. Therefore, redundancy is defined by the number of measurement units. The sensing precision of the device was subsequently assessed, with the results displayed in Figure 5. Figure 5a shows the deviation associated with the lateral sensing unit. With the test frequency band of 1–11 Hz, the module’s detection error consistently remained at a low level. When the frequency increased to 11 Hz, the error rose slightly to 2.7%. As shown in Figure 5b, the measurement error of the axial sensing module was slightly higher than that of the lateral module, but it also demonstrated reliable stability. Within the frequency range, its measurement error fluctuated within a narrow band and did not exhibit any significant trend with changes in frequency.

Both the lateral and axial modules of the sensor demonstrated excellent measurement accuracy and stability within their respective operating frequency ranges, with measurement errors consistently remaining below 3.5%. This indicates that the sensor can provide a reliable and precise response to external vibration frequencies, thus validating its feasibility as a high-precision vibration sensor.

**Figure 6:**
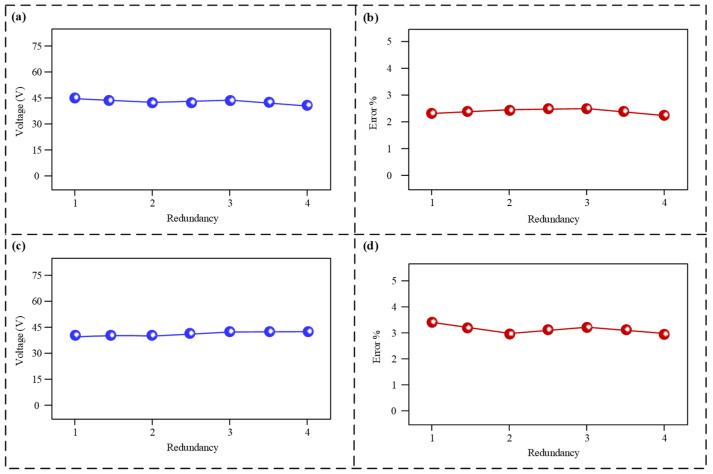
Redundancy test results. (**a**) Voltage output signals from the lateral module under various redundancy levels; (**b**) current signals generated by the lateral module across various redundancy levels; (**c**) voltage output signals from the axial module at varying redundancy levels; (**d**) current signals generated by the axial module at different redundancy levels.

To validate the reliability and fault tolerance of the sensor, its output performance and measurement error were tested under different levels of redundancy, with the results shown in Figure 6. Figure 6a,b present the test results for the lateral module at various redundancy levels. As the redundancy increased from 1 to 4 (i.e., the quantity of operational sensing units rose from one to four), the generated voltage amplitude remained stable within the range of approximately 43 V to 45 V. The measurement error consistently stayed below 2.5%, meeting practical engineering requirements. Figure 6c,d show the output signals from the axial vibration component across various redundancy levels. Its voltage output was maintained at around 43 V across all redundancy levels. The measurement error exhibited slight fluctuations but consistently remained below 3.5%.

These results indicate that both the lateral and axial modules of the sensor can provide stable voltage output and frequency measurements, regardless of whether a single sensing unit is active or multiple units are working collaboratively. This demonstrates that the failure of some sensing units will not affect the overall sensing performance, thereby effectively enhancing the sensor’s reliability over long-term use.

### 3.4. Power Generation Test Results

**Figure 7:**
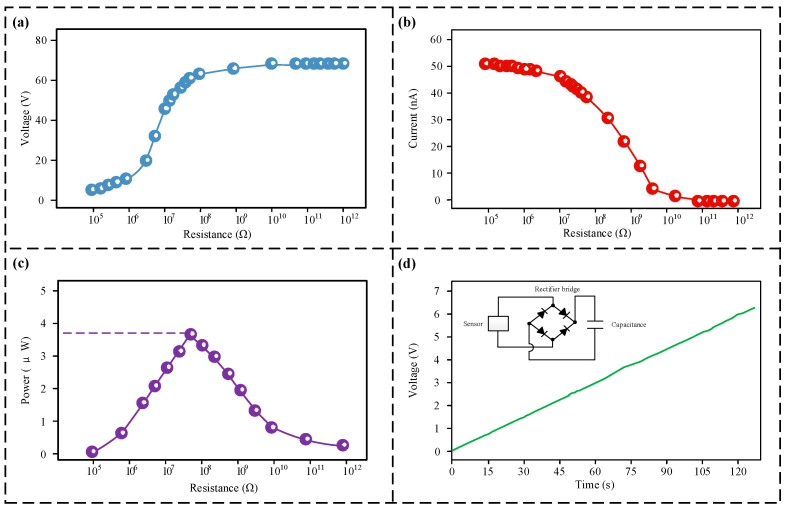
Evaluation of power generation. (**a**) Voltage generated by the sensor across various load resistances; (**b**) current response of the sensor to varying loads; (**c**) power output characteristics of the sensor relative to load changes; (**d**) assessment of the device’s charging ability.

The device operates on the fundamental mechanism of a triboelectric nanogenerator, meaning its function is intrinsically tied to triboelectric energy harvesting. Consequently, an assessment of the sensor’s energy generation potential was conducted, as illustrated in Figure 7. According to Figure 7a,b, an elevation in external load resistance causes the output voltage to rise progressively, whereas the output current shows a gradual decline. This trend adheres to Ohm’s law, with maximum potential and current intensity measured to be 68 V and 51 nA, respectively. Figure 6c illustrates a non-linear relationship linking the power generated by the device and the applied load resistance, attaining a maximum power output of roughly 3.8 × 10^−7^ at a load of 6 × 10^7^ Ω. As displayed in Figure 6d, data from a trial involving the charging of a 1 μF capacitor using the generated energy are provided. The voltage climbed to 6.2 V over a 120 s charging period, demonstrating that the device possesses efficient capability for charging.

### 3.5. Operating Condition Adaptability Test Results

**Figure 8:**
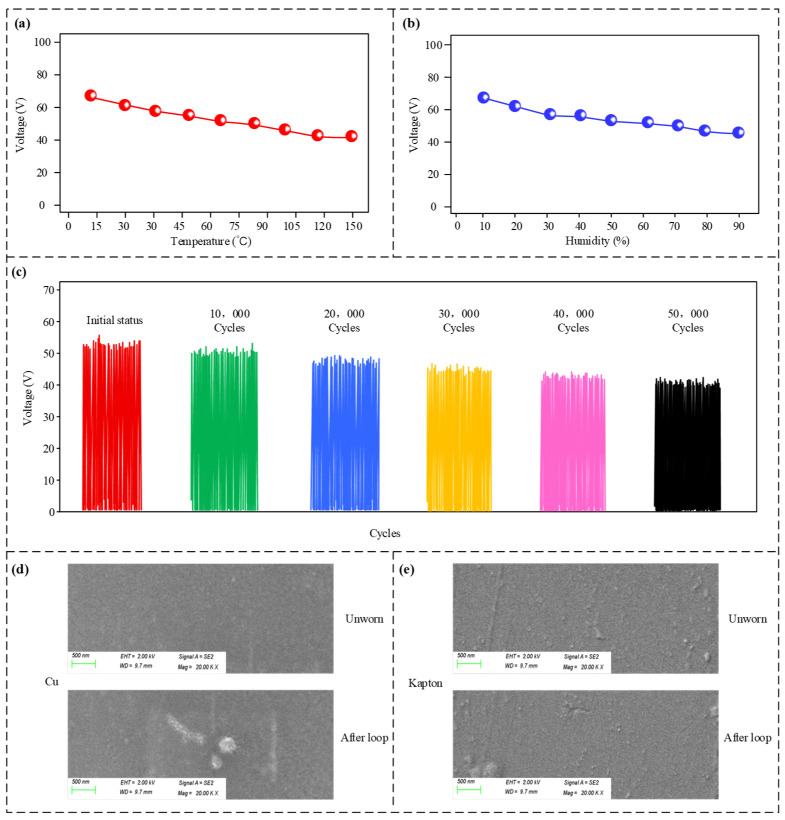
Operating condition adaptability test results. (**a**) Output voltage at different temperatures; (**b**) output voltage at different relative humidities; (**c**) output voltage of the sensor after different numbers of operating cycles. (**d**) SEM image of copper; (**e**) SEM image of Kapton.

Tests regarding operational adaptability were performed to assess the sensor’s stability and robustness under diverse drilling conditions, with findings illustrated in Figure 8. Figure 8a indicates that, within a temperature span of 0 °C to 150 °C, an inverse relationship exists between temperature and output voltage. The decrease in the sensor’s output voltage signal at high temperatures is also partly related to the material. The 3D-printed parts of the sensor are made from modified PLA material, which boasts a heat resistance exceeding 150 °C. However, at elevated temperatures, slight internal deformation may occur, leading to inconsistency. Likewise, Figure 8b reveals that rising humidity (10–90%) leads to a reduction in voltage output. Nevertheless, the voltage consistently stayed above 40 V across the full testing spectrum. Figure 8c examines the voltage output relative to the number of operation cycles. Although the voltage amplitude declined as cycles accumulated, it maintained a level of roughly 44 V after 50,000 cycles.

The sensor generates a pulsed output signal. This signal is directly fed into the microcontroller’s pulse input port, which is responsible for counting the number of pulses within a given timeframe. The pulse input port generally conforms to the TTL (Transistor–Transistor Logic) standard, where an input signal exceeding 2 Volts is interpreted as a logic high. Consequently, if the voltage is greater than 2 V, the microcontroller registers a ‘1’; otherwise, it registers a ‘0’. The data suggests that despite attenuation caused by high heat, humidity, and extended use, the sensor’s minimum output exceeds the 2 V TTL limit. Thus, downstream signal processing remains unaffected, highlighting the sensor’s strong environmental compatibility and extended lifespan. Figure 8d,e show the surface morphology of copper and Kapton before and after 50,000 cycles, respectively. After 50,000 cycles, the copper surface showed obvious scratches and some wear particles, while the Kapton surface showed only slight scratches and surface roughening, with significantly less wear than copper, indicating better mechanical durability.

### 3.6. Comparison of Sensor Types

After testing, we conducted a comparative study on various vibration sensors. As shown in Table 1, traditional chip-based vibration sensors offer high measurement accuracy, but they lack power generation capabilities, requiring a continuous external power supply. Furthermore, their design lacks high redundancy, making them prone to damage in strong vibration environments. Other TENG-based vibration sensors, while possessing power generation capabilities, often suffer from insufficient redundancy. This can easily lead to data loss if a sensing unit malfunctions. Moreover, replacing these sensors necessitates a tripping operation (pulling the drill string out of the well), which severely impacts drilling efficiency and economic benefits. In contrast, the sensor developed in this study boasts higher redundancy and integrates a self-powering function, making it more suitable for actual downhole environments.

## 4. Conclusions

Based on the principle of a triboelectric nanogenerator, this research details the creation of a redundant vibration monitoring device for downhole applications, designed to detect both axial and transverse movements. It is indicated by the test results that the operational frequency span of the unit is from 0 to 11 Hz, maintaining a measurement error of less than 3.5%. The device also functions reliably in environments with temperatures up to 150 °C and humidity levels up to 90%, proving its strong environmental resilience. Moreover, the device features self-powering attributes, generating a maximum potential reaching 68 V and a current intensity of 50 nA. A maximum power generation of 3.8 × 10^−7^ W is attained when the external load resistance is set to 6 × 10^7^ Ω.

This device presents two primary benefits. Firstly, the self-powering capability significantly mitigates or eliminates the dependence of subsurface sensors on external power sources, thereby significantly reducing the risk of operational interruptions caused by power depletion or line failures, which, in turn, saves operational expenses and enhances drilling productivity. Secondly, the device features a high-reliability redundant monitoring capability. It can simultaneously measure both axial and transverse vibrations, and even if one of the measurement units is damaged and becomes inoperable, the remaining sensing unit can continue to function normally, effectively enhancing the overall system’s reliability and risk resilience.

Although the sensor is self-powered, its current power output is still relatively small, making it unable to serve as an autonomous energy supply for other real-time MWD (measurement while drilling) units downhole. That is to say, the sensor can currently only be used as a self-powered sensor and cannot yet function as a power source. In the future, research ought to be conducted in the following two areas to enhance its energy production: First, the device’s architecture should be improved to enable it to simultaneously capture energy from multiple mechanical sources, such as bottomhole vibrations, mud circulation, and the rotation of the drill bit. This approach would expand the range of energy origins and boost the overall output power. Secondly, novel triboelectric materials with higher surface charge density should be developed. By constructing micro-structured arrays upon the friction layer surfaces, the contact area and charge transfer efficiency can be effectively enhanced, and this would fundamentally improve the sensor’s power density and energy conversion efficiency. Furthermore, subsequent signal processing circuits can be studied to further improve sensor accuracy.

## Figures and Tables

**Table 1:** Comparison of different types of sensors.

Sensor Types	Error Rate	Redundancy	Generated Power
This work	4%	4 channels	3.8 × 10^−7^ W
Chip-based	0.6%	None	None
LV-TENG	3%	None	1.8 × 10^−7^ W
Ball-TENG	5%	None	4.6 × 10^−6^ W

## Data Availability

The original contributions presented in the study are included in the article. Further inquiries can be directed to the corresponding author.
